# Together for health: introducing the International Foundation for Integrated Care

**DOI:** 10.5334/ijic.1126

**Published:** 2012-12-24

**Authors:** Nick Goodwin, Lourdes Ferrer

**Affiliations:** The King’s Fund, UK; International Foundation for Integrated Care, UK

## Together for health: introducing the International Foundation for Integrated Care

Integrated care is now a recognized paradigm for health care reforms around the globe. The need to address the problem of fragmented systems of care that undermine the performance of health care systems and result in poor quality care and outcomes to people has become a key policy ambition in many countries and regions. For example, in the Americas, resolutions have been passed in support of integrated health services delivery networks to address the adverse consequences of health service fragmentation [1]. Incoming governments in England, Belgium and the Netherlands have strongly promoted integrated care as a central theme in their health reform agendas (e.g. [2]). In the USA, the Affordable Care Act and the subsequent piloting of Accountable Care Organisations has sought to promote care co-ordination as well as boost health care coverage [3]. Specific agencies have even been developed, such as the Agency for Integrated Care in Singapore, tasked with developing and implementing its national agenda for integrated care [4].

Over the past few years, we have noticed the rise in the numbers of academic papers that have been submitted to *IJIC* and an incremental growth in the quality of the concepts, methods and research studies being reported. Our issues from the last two years, for example, contain far more research and theory articles than in previous years. Such a rise in the volume and quality of submissions to *IJIC* almost certainly reflects the increasing political interest in integrated care, and the subsequent rise in funding available for research.

Nonetheless, a content analysis of our Journal shows that more than 70% of our papers remain focused on the effectiveness of specific integration processes (such as partnership working, case management or clinical networks) with relatively few examining whole system changes or any demonstrable effect in terms of long-term impact. Much of the central hypothesis of integrated care—that the approach should improve people’s care experiences and outcomes cost-effectively—is intuitive but we need to learn more about how care systems can really adapt to embed the core properties of integrated care. It is fitting, therefore, that we welcome in this editorial the creation of the International Foundation for Integrated Care (IFIC)—a not-for-profit Foundation registered in the Netherlands—and celebrate *IJIC*’s new role as its scientific periodical. Under the slogan ‘Together for Health’, IFIC’s central mission will be to bring people together in order to advance the science, knowledge and adoption of integrated care policy and practice. IFIC seeks to achieve this through the development and exchange of ideas among academics, researchers, managers, clinicians, policy-makers and users and carers of services throughout the World [5].

The interpretation of integrated care, as we know, varies considerably. A core priority for IFIC will be to develop as a central and authoritative voice in this international debate, facilitating and leading our understanding of integrated care and how it can be achieved to all who would benefit from it.

IFIC has a growing range of activities to support this mission. In addition to supporting this Journal, activities planned for 2013 include:

- **Annual Conference** [6]: The 13^th^ edition of the conference will be held in Berlin, Germany, from the 11^th^ to 13^th^ April. It will examine four key challenges in integrated care: payment systems and incentives; continuity of care and care co-ordination; organisational solutions; and implementation strategies
- **World Congress** [7]: IFIC will be partnering with the Agency for Integrated Care (AIC) to support the first World Congress in Singapore between the 8^th^ and 9^th^ November 2013
- **Research and Development** [8]: IFIC is acting as a key partner within Project INTEGRATE, a four year EU-funded research project. The work seeks to look at the evidence for successful integrated care practices across four tracer conditions (diabetes, COPD, geriatrics and mental health) with a focus on the importance of number of cross-cutting issues such as patient engagement and involvement; care processes; human resource management and skill mix; information technology and communication; and financial flows. The research will develop much needed evidence into the necessary managerial and leadership practices that can best support integrated care, as well as key lessons for policy-makers.
- **Events and Study Tours** [9]: IFIC will be developing a European-based study tour programme (linked to Project INTEGRATE) and will again by supporting the successful International Congress on Telehealth and Telecare to be held in London on 1^st^–3^rd^ July 2013.
- **Education and Learning** [10]: IFIC has plans to develop a range of on-line learning resources over the coming year, including the creation of its first summer school programme. An Observatory in Integrated Care is planned, an initiative that seeks to develop a centre of knowledge, information and expertise on integrated care.

IFIC will develop as a membership-based organisation of individuals, institutions and networks that seek to join this rewarding effort of advancing the knowledge and practice in integrated care. Registration to become a member of IFIC is now open [11]. See Table 1 for membership fees and types.

We hope that you will join the International Foundation for Integrated Care and, in so doing, help to contribute to both the continued success of our Journal as well as the advancement of the science and knowledge of integrated care around the World. To join create a free account at the IFIC website and then register as an IFIC member at: http://www.integratedcarefoundation.org/content/membership.

## Figures and Tables

**Table 1.**
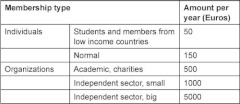
IFIC membership types and fees.

## References

[1] Pan American Health Organisation (2011). Integrated health service delivery networks: concepts, policy options and a road map for implementation in the Americas.

[2] Department of Health (2012). Integration. A report from the NHS Future Forum.

[3] Shortell SM, Weinberger S (2012). Advancing capabilities of safety net accountable care organizations. Policy brief.

[4] Cheah J (2011). Integrate now, create health. International Journal of Integrated Care [serial online].

[5] International Foundation for Integrated Care Homepage. [Webpage on the internet]. [cited 2012 Nov 30]. Available from: http://www.integratedcarefoundation.org.

[6] International Foundation for Integrated Care Annual conferences. [Webpage on the internet]. [cited 2012 Nov 30]. Available from: http://www.integratedcarefoundation.org/conferences.

[7] International Foundation for Integrated Care World congresses. [webpage on the internet] [cited 2012 Nov 30]. Available from: http://www.integratedcarefoundation.org/content/world-congresses.

[8] International Foundation for Integrated Care Research and development. [Webpage on the internet]. [Cited 2012 Nov 30]. Available from: http://www.integratedcarefoundation.org/content/research-development.

[9] International Foundation for Integrated Care Events and study tours. [webpage on the internet]. [Cited 2012 Nov 30]. Available from: http://www.integratedcarefoundation.org/content/events-study-tours.

[10] International Foundation for Integrated Care Education and learning. [Webpage on the internet]. [Cited 2012 Nov 30]. Available from: http://www.integratedcarefoundation.org/content/education-learning.

[11] International Foundation for Integrated Care Members network. [Webpage on the internet]. [Cited 2012 Nov 30]. Available from: http://www.integratedcarefoundation.org/content/membership.

